# Proteome analysis of bronchoalveolar lavage in pulmonary langerhans cell histiocytosis

**DOI:** 10.1186/2043-9113-1-31

**Published:** 2011-11-10

**Authors:** Claudia Landi, Elena Bargagli, Barbara Magi, Antje Prasse, Joachim Muller-Quernheim, Luca Bini, Paola Rottoli

**Affiliations:** 1 Respiratory Diseases Section, Department of Clinical Medicine and Immunological Sciences, University of Siena, Siena (Italy; 2 Department of Biotechnologies, University of Siena, Siena (Italy; 3 Department of Pneumology, Ludwig University, Freiburg (Germany

## Abstract

**Background:**

Pulmonary Langerhans-cell histiocytosis (PLCH) is a rare interstitial lung disease characterized by clusters of Langerhans cells, organized in granulomas, in the walls of distal bronchioles. It is a diffuse lung disease related to tobacco smoking but otherwise of unknown etiopathogenesis.

**Methods:**

In this study we used a proteomic approach to analyze BAL protein composition of patients with PLCH and of healthy smoker and non-smoker controls to obtain insights into the pathogenetic mechanisms of the disease, to study the effect of cigarette smoking on susceptibility to PLCH and to identify potential new biomarkers.

**Results:**

Two-dimensional electrophoresis and image analysis revealed proteins that were differently expressed (quantitatively and qualitatively) in the three groups of subjects. The proteins were identified by mass spectrometry and have various functions (antioxidant, proinflammatory, antiprotease) and origins (plasma, locally produced, etc.). Many, such as protease inhibitors (human serpin B3) and antioxidant proteins (glutathione peroxidase and thioredoxin) are already linked to PLCH pathogenesis, whereas other proteins have never been associated with the disease. Interestingly, numerous proteolytic fragments of plasma proteins (including kininogen-1 N fragments and haptoglobin) were also identified and suggest increased proteolytic activity in this inflammatory lung disease. Differences in protein expression were found between the three groups and confirmed by Principal Component Analysis (PCA).

**Conclusion:**

Analysis of BAL proteomes of PLCH patients and of smoker and non-smoker controls also proved to be useful for researching the pathogenetic mechanisms and for identifying biomarkers of this rare diffuse lung disease.

## Introduction

Pulmonary Langerhans cell histiocytosis (PLCH) is a rare granulomatous disorder characterized by uncontrolled proliferation and infiltration of CD1+ Langerhans cells (LCs) in the lung. It has been associated with smoking and prevalently affects young adults [1,2]. The pathogenesis of PLCH is unclear. The bronchiolar distribution of lesions suggests that an inhaled antigen, such as cigarette smoke, may be involved, since 90% of cases are smokers [3]. The correlation between PLCH and smoking is corroborated by recent studies demonstrating that acute tobacco smoke inhalation determines immediate and selective recruitment of LCs into human airways, inducing a very early reaction of the adaptive immune system [4-6]. Moreover, cigarette smoke promotes survival signals and prolongs survival of dendritic cells [7]. Smoke-induced alterations at lung level can therefore induce changes in lung condition determining a typical protein profile at bronchoalveolar and plasma level.

Proteomics is a powerful approach that enables lung diseases to be studied through the characterization and identification of protein marker profiles that can highlight specific pathological states. A proteomic approach to the study of BAL is extremely useful for insights into pathogenesis and identification of biomarkers [8]. There is no literature on BAL proteomic findings in PLCH. We therefore studied BAL protein composition in PLCH patients, healthy non-smoker controls and healthy smoker controls by a proteomic approach using two-dimensional electrophoresis (2-DE) and mass spectrometry (MS) in order to obtain insights into the pathogenesis of PLCH, to evaluate the effect of smoking on disease progression and to discover new prognostic biomarkers.

## Materials and methods

### Population

The study population consisted of five PLCH patients of Caucasian race (3 female, mean age 33.15 ± 36.13 years), five healthy non-smokers (3 female, mean age 59.13 ± 24.2) and five healthy smokers (2 female, mean age 43.17 ± 29.62) monitored at Siena Regional Referral Centre for Interstitial Lung Diseases for a period of at least four years. All patients were currently smokers with the exception of a single patient who was an ex-smoker. We analyzed exposure of our patients to environmental pollution retrospectively and interestingly, none of the patients lived in big cities: all came from the country or small towns with no significant exposure to pollutants. No professional risk was found as 3/5 were office workers, another a teacher and the fifth a cook. Diagnosis of PLCH was conducted according to international criteria [9-11]; three patients had a diagnosis based on histological examination of transbronchial biopsies showing tissue positivity for anti-CD1a and S100 protein staining; the other two had a diagnosis based on clinical-radiological findings and BAL features (including CD1a positivity). All patients underwent pulmonary function tests (PFT) and gas exchange evaluation according to ERS guidelines [12]. All patients gave their written informed consent to enrolment in the study.

### Bronchoalveolar lavage

Bronchoscopy with BAL was performed in all patients for diagnostic reasons as previously reported [13-15]. Lymphocyte phenotype was analyzed by flow cytometry (Facs-Calibur, Becton Dickinson) using anti -CD3, -CD4, -CD8 and -CD1a monoclonal antibodies.

### Two-Dimensional Gel Electrophoresis (2DE)

BAL samples were dialyzed against water, lyophilized and dissolved in lysis buffer (8 M urea, 4% CHAPS, 40 mM Tris base, 65 mM dithioerythritol and trace amounts of bromophenol blue). Protein concentration was determined according the Bradford method [16]. 2DE was carried out using the Immobiline polyacrylamide system, as previously described [17] on a preformed immobilized nonlinear pH gradient, from pH 3 to 10, 18 cm length, from GE Healthcare (Uppsala, Sweden). Sample load was 60 μg per strip in analytical runs, and 1 mg per strip in preparative gels. Analytical runs were carried out using the Ettan™ IPGphor™ system (Amersham Biosciences) at 16°C under the following electrical conditions: 0 V for 1 h, 30 V for 8 h, 200 V for 1 h, from 300 to 3500 V in 30 min, 3500 V for 3 h, from 3500 to 8000 V in 30 min, 8000 V up to a total of 80,000 Vh. Preparative strips were rehydrated with 350 μL UREA 8 M, 4% w/v CHAPS, 1% w/v DTE and 2% v/v carrier ampholyte at room temperature for 12 h. Sample load was obtained by cup loading, with the cup applied at the cathodic and anodic ends of the strip. MS-preparative runs were obtained using the Multiphor™ II electrophoresis system and the following voltage steps at 16°C: 200 V for 6 h, 600 V for 1 h, 1200 V for 1 h, 3500 V for 3 h, 5000 V for 14 h. After the first dimension run, the IPG gels were equilibrated in 6 M urea, 2% w/v SDS, 2% w/v DTE, 30% v/v glycerol and 0.05 M Tris-HCl pH 6.8 for 12 min; and for a further 5 min in 6 M urea, 2% w/v SDS, 2.5% w/v iodoacetamide, 30% v/v glycerol, 0.05 M Tris-HCl pH 6.8 and a trace of bromophenol blue. After the two equilibration steps, the second dimensional separation was performed on 9-16% SDS polyacrylamide linear gradient gels (18 × 20 cm × 1.5 mm), and carried out at 40 mA/gel constant current, at 9°C until the dye front reached the bottom of the gel [18]. Analytical gels were stained with ammoniacal silver nitrate [19,20]. MS-preparative gels were stained with SYPRO Ruby (Bio-rad headquarters, Hercules, California) according to the manufacturer's instructions. Bind-silane (γ methacryloxypropyltrimethoxysilane) (LKBProdukter AB, Brommo, Sweden) was used to attach polyacrylamide gels covalently to a glass surface for those undergoing SYPRO Ruby staining [21]. Ammoniacal silver nitrate stained gels were then digitized by a Molecular Dynamics 300S laser densitometer (4000 × 5000 pixels, 12 bits/pixel; Sunnyvale, CA, USA). Preparative gel images stained with SYPRO Ruby were digitized with a Typhoon 9400 laser densitometer (GE Healthcare). Computer-aided 2D image analysis was carried out with the Image Master Platinum 7.0 computer system (GE Healthcare). Spot detection was achieved after defining and saving a set of detection parameters, enabling filtering and smoothing of the original gel scans to clarify spots, and removal of vertical and horizontal streaks and speckles. The analysis process was performed by matching all gels of each group with a reference gel for the same condition with the best resolution and greatest number of spots, chosen by the user and named "master" by the software. The three master reference gels were then matched with each other. By this procedure, the Image Master Platinum algorithm matched the other gels to find qualitative and quantitative differences.

### Statistical analysis

Statistical analysis of the samples was performed using Statistical software packages SPSS 13.0 for Windows and Graphpad Prism 5 for Windows. Data was expressed as mean ± standard deviation (M ± SD). For the proteomic approach, statistical analysis of proteins expressed differently in the three groups was carried out using Student's T-test, one-way ANOVA and Tukey's test. Only unmatched spots or spots with significantly different %V (p < 0.05 by ANOVA) were considered "differently expressed" in the three groups.

### Mass Spectrometry

Protein identification was carried out by PMF on an Ettan MALDI-TOF Pro (GE Healthcare), as previously described [22,23]. Electrophoretic spots from SYPRO Ruby stained gels were mechanically excised by an Ettan Spot Picker (GE Healthcare), destained in 2.5 mM ammonium bicarbonate and 50% acetonitrile, and dehydrated in acetonitrile. They were then rehydrated in trypsin solution and digested overnight at 37°C. 0.75 μL of each protein digest was spotted onto the MALDI target and allowed to dry. Then 0.75 μL of matrix solution (saturated solution of CHCA in 50% v/v ACN and 0.5% v/v TFA) was applied to the dried sample, and dried again. After acquiring the mass of the peptide, a mass fingerprinting search was carried out in Swiss-Prot/TrEMBL and NCBInr databases using MASCOT (Matrix Science Ltd., London, UK, http://www.matrixscience.com) software available on-line. Taxonomy was limited to Mammalia, mass tolerance was 100 ppm, and the number of missed cleavage sites accepted was set at one. Alkylation of cysteine by carbamidomethylation was assumed and oxidation of methionine was considered as a possible modification. Sequence coverage, number of matched peptides and probability score are shown in the tables.

### Multivariate analysis

Principal Components Analysis (PCA) was performed for the three groups to reduce proteomic data complexity and to identify meaningful groups and associations in the dataset. PCA transforms a number of correlated variables (e.g. individual protein spot abundance levels in each experimental sample) into a smaller number of uncorrelated variables, called principal components. In this study PCA was used to cluster the experimental groups on the basis of protein spot expression in BAL (spot maps). Percentage volumes of spots differently expressed in the three analysis groups (PLCH *versus *non-smoker controls, PLCH *versus *smoker controls and non-smoker *versus *smoker controls) were included in the PCA analysis, which was performed using STATISTICA 7.0 software (Statsoft, Inc.). In the resulting graph, the spot maps were plotted in two-dimensional space, showing the principal components PC1 and PC2 that divided the samples analyzed orthogonally according to the two principal sources of variation in the data set.

## Results

### Population

Table 1 reports the clinical features, LFT and bronchoalveolar lavage results of the group of PLCH patients. As expected, BAL cell profile showed eosinophilia greater than 6%, mild neutrophilia and 8.1% [± 5.3] CD1a-positive cells. Low DLCO was evident in all patients at the time of bronchoscopy and lung function tests revealed obstructive pattern in 2 patients, restrictive deficit in 1 patient and a normal functional pattern in the other 2 cases.

**Table 1:** Clinical characteristics, BAL and LFT features of our PLCH population.

NUMBER OF PATIENTS	5
**AGE**	33,15 ± 36,13

**GENDER**	2 MALE

**BAL MACROPHAGES (%)**	77,2 ± 15,6

**BAL LYMPHOCYTES (%)**	9,9 ± 19,3

**BAL NEUTROPHILS (%)**	4,7 ± 2,1

**BAL EOSINOPHILS (%)**	6,88 ± 3,4

**CD1+**	8,1 ± 5,3

**OBSTRUCTIVE**	2

**RESTRICTIVE**	1

**DECREASED** **DLCO**	5

### Proteome analysis

**Figure 1:**
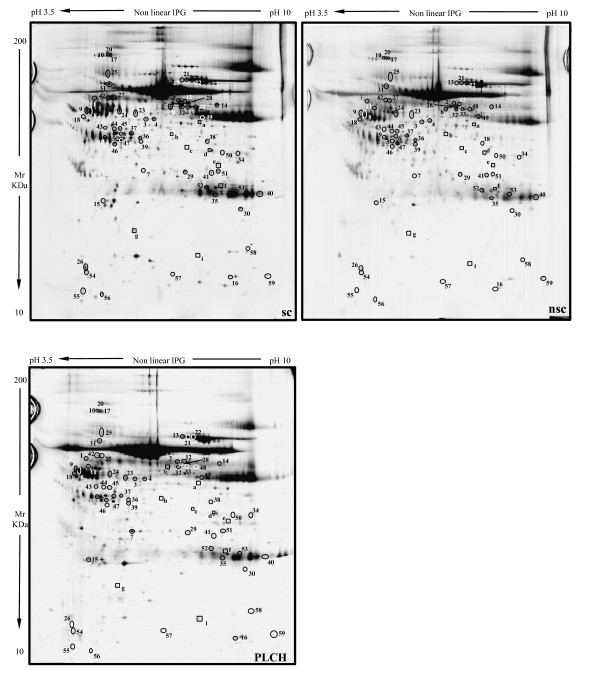
**The master gels of the three groups: PLCH patients and smoker/non-smoker controls**.

Figure 1 shows the master gels of the three groups (PLCH patients and smoker/non-smoker controls), chosen as reference gels because of their high resolution and large number of protein spots. An average of 1100 spots was detected in each gel across groups. When our master gels were matched by Image Master Platinum 7.0, qualitative and quantitative protein differences were observed. MALDI-ToF/MS identified these proteins, including two found for the first time in BAL samples: serpin B3 (SPB3) and plastin-2 (PLSL), which were up-regulated in smokers versus non-smokers and down-regulated in PLCH patients versus smokers. Among spots expressed differently between groups, there were modulators of immune responses (such as polymeric immunoglobulin receptor (PIGR), immunoglobulin light chain, Ig alpha-1 chain C region, PLSL, Ig gamma-1 chain C region, IgG K chain), proteins implicated in antioxidant defence (thioredoxin (THIO), albumin (ALBU), ceruloplasmin (CERU), glutathione peroxidase 3 (GPX3)), cell-cycle regulators (creatinine kinase B-Type, ADP ribosylation factor-like protein 3 and annexin A3 (ANXA3)), proteins involved in ion transport (such as serotransferrin (TRFE) and hemoglobin subunit beta) and several inflammatory proteins (including pigment epithelium derived factor (PEDF) and apolipoprotein A1 (APOA1)). Alpha-1-antitrypsin (A1AT) isoforms and SPB3 were spots with anti-protease function. Other proteins like purine nucleoside phosphorylase, pyruvate kinase isozymes, fibrinogen gamma chain, alpha 1B glycoprotein and actin cytoplasmic 1 were identified. BAL proteome analysis of PLCH patients also revealed several proteolytic fragments of plasma proteins, such as albumin (ALBU), haptoglobin (HPT) and kininogen-1 (KNG1). Five isoforms of alpha 1 anti-trypsin (A1AT) were differentially expressed in BAL of the three groups.

Considering only spots constantly present in all gels of all groups, significant qualitative variations in sensitivity to silver staining were observed for the nine spots (Table 2). Some of these proteins were found in healthy controls but not in patients and others were found in PLCH and smoker-control samples but not in those of non-smoker controls. Fifty nine spots showed at least ± 2 times variations in percentage of relative volume (%V) (%V = Vsingle spot/Vtotal spot). These spots were significantly up- or down-regulated in BAL samples of PLCH patients with respect to BAL of smoker and non-smoker controls (p < 0.05). Tables 3, 4 and 5 list the proteins identified from these spots with their accession numbers, theoretical and experimental molecular weights, p*I*s, Mascot search results, mean and standard deviations, statistical *p *values and number of folds of protein expression in the three groups.

**Table 2:** Proteins identified in BAL qualitatively differently expressed in PLCH patients than controls.

Spot letter	Protein name	AC	Theoretical pI/Mr (KDa)	Experimental pI/Mr (KDa)	Mascot search result			Mean %V ± SD × 10^-4^			1-way ANOVA *p *value	Localization
							
					No. of matched peptide	Sequence coverage	Score	nsc	sc	PLCH		
a	Serum albumin, fragment c-term	P02768	5.92	6.3	5	11	64	67**±**68*	175**±**56*^¥^	0**^¥^**	0,0005	Plasma
			71317	47485								

B	Serum albumin, fragment c-term	P02768	5.92	5.9	10	20	128	28**±**39*	125**±**69*^¥^	0**^¥^**	0,002	Plasma
			71317	41761								
												

D	Alcohol dehydrogenase	P14550	6.32	6.44	15	52	223	90**±**101*	396**±**159*^¥^	0**^¥^**	0,0002	Cytoplasm
			36892	37399								

e	Annexin A1	P04083	6.57	6.57	12	42	179	22**±**35*	326**±**265*^¥^	0**^¥^**	0,009	Cytoplasm-Nucleus
			38918	33339								

g	Glutathione peroxidase 3	P22352	8.26	5.48	9	34	121	108**±**40*	386**±**229*^¥^	0**^¥^**	0,001	Plasma
			25765	17509								

h	Beta-2-glycoprotein 1	P02749	8.34	5.89	6	28	91	0*	997**±**277*^¥^	319**±**253**^¥^**	3,27E-05	Plasma
			39584	56152								

i	Serum albumin, fragment c-term	P02768	5.92	6.25	8	11	78	0**^§^**	61**±**97**^¥^**	381**±**254**^¥§^**	0,005	Plasma
			71317	14042								

**Table 3:** List of identified proteins with their accession numbers, theoretical and experimental molecular weights, p*I*s,

No. of spot	Protein name	AC	Theoretical pI/Mr (KDa)	Experimental pI/Mr (KDa)	Mascot search result			Mean %V ± SD × 10-4			1-way ANOVA p value	Folds in			Localization
								
					No. of matched peptide	Sequence coverage (%)	Score	nsc	sc	PLCH		Nsc-sc	Nsc-PLCH	Sc-PLCH	
**PLCH > nsc and/or sc**															

**1**	Kininogen-1, fragment N-term	P01042	6.34	4.95	11	18	118	543**±**105**^§^**	225**±**214**^¥^**	5393**±**1864**^¥§^**	8.97E-06	2.41	9.93**^§^**	23.96**^¥^**	Secreted-extracellular space

			72996	61092											

**2**	Ig alpha-1 chain C region	**P01876**	6.08	5.98	8	25	116	879**±**788**^§^**	206**±**207**^¥^**	3179**±**1685**^¥§^**	0,002	4.26	3.61**^§^**	15.43**^¥^**	Secreted

			38486	60221											

**3**	Pigment epithelium	P36955	5.97	5.61	8	24	111	405**±**176**^§^**	281**±**87**^¥^**	1230**±**693**^¥§^**	0,007	1.44	3.03**^§^**	4.37**^¥^**	Secreted

			46484	49075											

**4**	Pigment epithelium	P36955	5.97	5.69	8	21	114	326**±**211**^§^**	307**±**182**^¥^**	767**±**260**^¥§^**	0,009	1.06	2.35**^§^**	2.49**^¥^**	Secreted

			46484	49222											

**5**	Haptoglobin, fragment c-term.	P00738	6.13	5.26	12	31	114	446**±**69**^§^**	432**±**180**^¥^**	2062**±**1186**^¥§^**	0,003	1.03	4.62**^§^**	4.77**^¥^**	Plasma

			45861	40355											

**6**	Creatine kinase B-type	**P12277**	5.34	5.31	7	26	108	267**±**146**^§^**	161**±**93**^¥^**	749**±**306**^¥§^**	0,001	1.65	2.80**^§^**	4.65**^¥^**	Cytoplasm

			42902	41841											

**7**	Annexin A3	**P12429**	5.63	5.59	8	32	111	158**±**159**^§^**	127**±**54**^¥^**	530**±**318**^¥§^**	0,017	1.24	3.35**^§^**	4.17**^¥^**	Cytoplasm

			36524	31681											

**8**	Alpha-1 anti tripsin	**P01009**	5.37	4.84	15	46	212	1491**±**1178**^§^**	299**±**413**^¥^**	5425**±**817**^¥§^**	1,79E-06	4.98	3.63**^§^**	18.14**^¥^**	Plasma

			46878	53626											

**9**	Alpha-1 anti tripsin	**P01009**	5.37	4.8	8	27	107	495 ± 979^§^	28 ± 64**^¥^**	2644 ± 1473^**¥§**^	0,003	17.6	5.34**^§^**	94.42**^¥^**	Plasma

			46878	54359											

**10**	Ceruloplasmin	P00450	5.44	5.1	11	13	134	135**±**118**^§^**	154**±**149**^¥^**	496**±**121**^¥§^**	0,001	1.14	3.67**^§^**	3.22**^¥^**	Plasma

			122983	131973											

**11**	Alpha-1-antitrypsin	**P01009**	5.37	4.96	13	41	175	288**±**221**^§^**	423**±**204**^¥^**	1359**±**551**^¥§^**	0,001	1.46	4.71**^§^**	3.21**^¥^**	Plasma

			46878	58032											

**12**	Serotransferrin	**P02787**	6.81	6.05	8	11	102	415**±**322**^§^**	162**±**219**^¥^**	2655**±**1223**^¥§^**	0,0003	2.56	6.39**^§^**	16.38**^¥^**	Plasma

			79280	60048											

**13**	Serotransferrin	**P02787**	6.81	6	11	19	112	623**±**170**^§^**	672**±**576**^¥^**	3255**±**2316**^¥§^**	0,016	1.07	5.22**^§^**	4.84**^¥^**	Plasma

			79280	80091											

**14**	Pyruvate kinase isozymes	**P14618**	7.96	6.47	11	28	105	276**±**236**^§^**	642**±**290**^¥^**	1391**±**596**^¥§^**	0,003	2.32	5.03**^§^**	2.16**^¥^**	Cytoplasm-Nucleus

			58470	58174											

**15**	Apolipoprotein A-I	P02647	5.56	5.03	8	26	99	179**±**103**^§^**	276**±**197**^¥^**	924**±**371**^¥§^**	0,0009	1.54	5.16**^§^**	3.34**^¥^**	Plasma

			30759	23065											

**16**	Hemoglobin subunit beta	**P68871**	6.75	6.77	11	77	155	109 ± 131^§^	328 ± 123**^¥^**	650 ± 233^**¥**§^	0,001	3	5.96**^§^**	1.98**^¥^**	Blood

			16102	11120											

**17**	Ceruloplasmin	P00450	5.44	5.13	15	18	123	259**±**183**^§^**	180**±**179**^¥^**	564**±**99**^¥§^**	0,005	1.43	2.17**^§^**	3.13**^¥^**	Plasma

			122983	130654											

**18**	Alpha-1-antitrypsin	**P01009**	5.37	4.7	11	32	143	277**±**290	93**±**77**^¥^**	599**±**288**^¥^**	0,018	2.97	2.16	6.44**^¥^**	Plasma

			46878	50566											

**19**	Actin, cytoplasmic 1	**P60709**	5.29	5.07	9	28	107	867**±**437	320**±**272**^¥^**	1071**±**348**^¥^**	0,016	2.7	1.23	3.34**^¥^**	Cytoskeleton

			42052	40664											

**20**	Ceruloplasmin	P00450	5.44	5.16	10	11	101	306**±**223	201**±**151**^¥^**	537**±**137**^¥^**	0,02	1.52	1.75	2.67**^¥^**	Plasma

			122983	130000											

**21**	Serotransferrin	**P02787**	6.81	6.07	16	22	166	1085**±**228**^§^**	1831**±**559	4471**±**2939**^§^**	0,022	1.68	4.12**^§^**	2.44	Plasma

			79280	80.091											

**22**	Serotransferrin	**P02787**	6.81	6.14	18	23	188	2376**±**300**^§^**	3235**±**879	4969**±**1743**^§^**	0,01	1.36	2.09**^§^**	1.53	Plasma

			79280	79397											

**23**	Fibrinogen gamma chain	**P02679**	5.37	5.52	10	28	133	511**±**354**^§^**	766**±**671	2649**±**2055**^§^**	0,039	1.49	5.18**^§^**	3.45	Plasma

			52106	53481											

**24**	Alpha-1-antitrypsin	**P01009**	5.37	5.29	8	27	121	197**±**222**^§^**	641**±**651	1938**±**1200**^§^**	0,01	3.25	9.83**^§^**	3.02**^¥^**	Plasma

			46878	54009											

Mascot search results, mean and standard deviations, statistical *p *values and number of folds of protein expression in the three groups. Statistical significant differences between smoking controls (sc) and no-smoking controls (nsc) are visualized by *, statistical differences between smoking controls and Pulmonary Langerhans cell Histiocytosis (PLCH) are visualized by **^¥^**and significant differences occurring between no-smoking controls and Pulmonary Langerhans cell Histiocytosis are shown by **^§^**. The proteins quantitatively more abundant in PLCH than in non-smoker and/or smoker control samples are reported in table 3.

**Table 4:** Proteins down-regulated in PLCH compared to non-smoker and/or smoker controls.

No. of spot	Protein name	AC	Theoretical pI/Mr (KDa)	Experimental pI/Mr (KDa)	Mascot search result			Mean %V ± SD × 10^-4^			1-way ANOVA *p *value	Folds in			Localization
								
					No. of matched peptide	Sequence coverage (%)	Score	nsc	sc	PLCH		Nsc-sc	Nsc-PLCH	Sc-PLCH	
**PLCH < nsc and/or sc**															

**25**	Polymeric immunoglobulin receptor	P01833	5.58	5.14	10	18	129	2901**±**438**^§^**	3037**±**1038**^¥^**	911**±**694**^¥§^**	1.31E-03	1.04	3.18**^§^**	3.33**^¥^**	Cell membrane
			84429	87377											

**26**	Thioredoxin	P10599	4.82	4.67	6	40	82	665**±**113	1492**±**819**^¥^**	230**±**235**^¥^**	0,005	2.24	2.89	6.48**^¥^**	Cytoplasm-Secreted
			12015	12098											

**27**	Plastin-2	P13796	5.2	5.19	10	21	102	873**±**355	1272**±**320**^¥^**	453**±**386**^¥^**	0,01	1.45	1.92	2.80**^¥^**	Cytoplasm-Cytoskeleton-Cell junktion
			70815	61983											

**28**	Serum albumin	P02768	5.92	6.04	8	14	92	1830**±**647	2980**±**827**^¥^**	1239**±**1363**^¥^**	0,04	1.62	1.47	2.40**^¥^**	Plasma
			71317	57513											

**29**	Serum albumin, fragment	P02768	5.92	6.09	6	9	74	278**±**137	405**±**224**^¥^**	124**±**83**^¥^**	0,049	1.45	2.24	3.26**^¥^**	Plasma
	N-term		71317	31059											

**30**	ADP-ribosylation factor-like protein 3	P36405	6.74	7.37	6	52	107	305**±**161	621**±**331**^¥^**	142**±**49**^¥^**	0,012	2.03	2.14	4.37**^¥^**	Membrane
			20614	21404											

**31**	Alpha-1B-glycoprotein	P04217	5.58	5.16	8	22	109	1370**±**414**^§^**	1163**±**443	663**±**83**^§^**	0,022	1.17	2.06**^§^**	1.75	Plasma
			54809	75685											

**Table 5:** Proteins differently expressed between smoker and no-smoker controls and between PLCH patients and controls.

No. of spot	Protein name	AC	Theoretical pI/Mr (KDa)	Experimental pI/Mr (KDa)	Mascot search result			Mean %V ± SD × 10^-4^			1-way ANOVA *p *value	Folds in			Localization
								
					No. of matched peptide	Sequence coverage (%)	Score	nsc	sc	PLCH		Nsc-sc	Nsc-PLCH	Sc-PLCH	
**PLCH < nsc and/or sc**															

**25**	Polymeric immunoglobulin receptor	P01833	5.58	5.14	10	18	129	2901**±**438**^§^**	3037**±**1038**^¥^**	911**±**694**^¥§^**	1.31E-03	1.04	3.18**^§^**	3.33**^¥^**	Cell membrane
			84429	87377											

**26**	Thioredoxin	P10599	4.82	4.67	6	40	82	665**±**113	1492**±**819**^¥^**	230**±**235**^¥^**	0,005	2.24	2.89	6.48**^¥^**	Cytoplasm-Secreted
			12015	12098											

**27**	Plastin-2	P13796	5.2	5.19	10	21	102	873**±**355	1272**±**320**^¥^**	453**±**386**^¥^**	0,01	1.45	1.92	2.80**^¥^**	Cytoplasm-Cytoskeleton-Cell junktion
			70815	61983											

**28**	Serum albumin	P02768	5.92	6.04	8	14	92	1830**±**647	2980**±**827**^¥^**	1239**±**1363**^¥^**	0,04	1.62	1.47	2.40**^¥^**	Plasma
			71317	57513											

**29**	Serum albumin, fragment	P02768	5.92	6.09	6	9	74	278**±**137	405**±**224**^¥^**	124**±**83**^¥^**	0,049	1.45	2.24	3.26**^¥^**	Plasma
	N-term		71317	31059											

**30**	ADP-ribosylation factor-like protein 3	P36405	6.74	7.37	6	52	107	305**±**161	621**±**331**^¥^**	142**±**49**^¥^**	0,012	2.03	2.14	4.37**^¥^**	Membrane
			20614	21404											

**31**	Alpha-1B-glycoprotein	P04217	5.58	5.16	8	22	109	1370**±**414**^§^**	1163**±**443	663**±**83**^§^**	0,022	1.17	2.06**^§^**	1.75	Plasma
			54809	75685											

This table is divided in two parts: the first part includes protein spots significantly down-regulated in non-smoker compared to smoker controls; the second part includes spots up-regulated in non-smoker than smoker controls.

**Figure 2:**
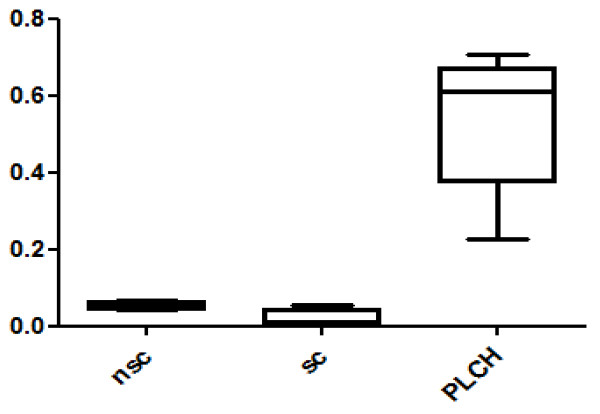
**KNG1 N-terminal fragment percentage of volume (%V) in BAL samples of PLCH patients and controls**.

**Figure 3:**
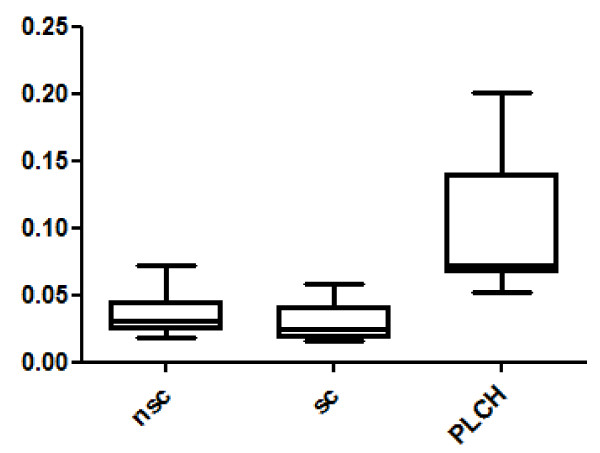
**The percentage of volume (%V) of two spots identified as PEDF in BAL samples of patients with PLCH respect to controls**.

**Figure 4:**
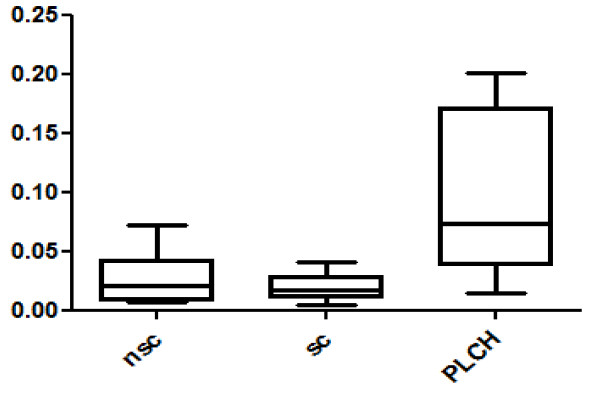
**Annexin A3 (ANXA3) %V in BAL of PLCH patients smoker controls and no-smoker controls**.

Twenty-eight spots were quantitatively more abundant in PLCH than in non-smoker and/or smoker control samples. The proteins of 24/28 spots were identified and are listed in Table 3. KNG1 fragment N-terminal (p < 0.00001) and an isoform of A1AT were strongly up-regulated in PLCH patients with respect to controls (Table 3). Figure 2 shows the expression of KNG1 N-terminal fragment (an inflammatory protein never studied in PLCH) in patients and controls. The percentage volume of two spots identified as PEDF (a protease inhibitor) were particularly elevated in patients than controls (p < 0.001) (Figure 3). Another protein involved in cell proliferation, motility, invasiveness and signaling pathways, up-regulated in PLCH with respect to controls (p < 0.01) and potentially involved in pathogenesis, is ANXA3 (Figure 4).

**Figure 5:**
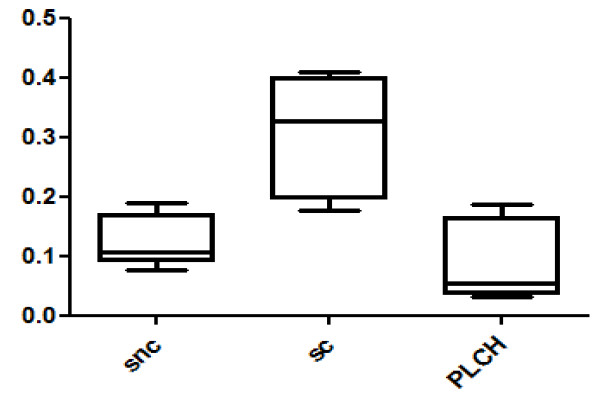
**Polymeric Immunoglobulin Receptor (PIGR) %V in BAL of PLCH patients smoker controls and no-smoker controls**.

**Figure 6:**
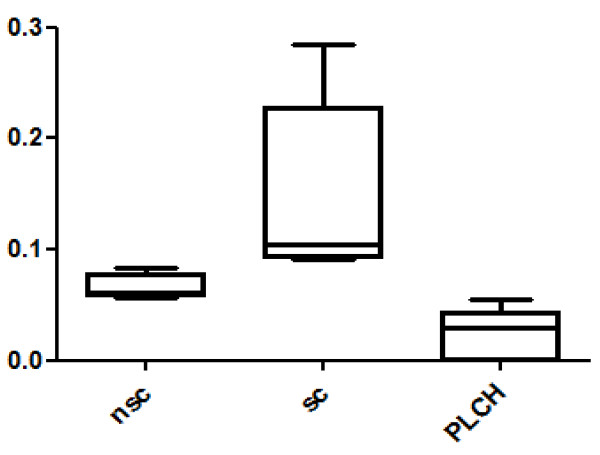
**Thioredoxin (THIO) in BAL samples of PLCH patients compared with smoker controls and no-smoker controls**.

Thirteen spots were down-regulated in PLCH compared to non-smoker and/or smoker controls (Table 4). The protein spots PIGR, THIO and PLSL were down-regulated in PLCH compared to controls and are of particular interest because of their specific functions and potential implication in the disease. Figures 5 and 6 show the trend of expression of PIGR, THIO percentage volumes in patients and controls.

Seventeen spots were also significantly differently expressed between healthy smoker and non-smoker controls, as well as between controls and PLCH patients; 10/17 were identified (table 5). Table 5 is divided in two parts: the first includes protein spots significantly down-regulated in non-smoker compared to smoker controls; the second includes spots up-regulated in non-smoker compared to smoker controls. Among the spots up-regulated in smokers, SPB3 is a protein with anti-protease function identified *de novo *in BAL; there is no literature on SPB3 and smoke-induced lung damage.

### Multivariate analysis

**Figure 7:**
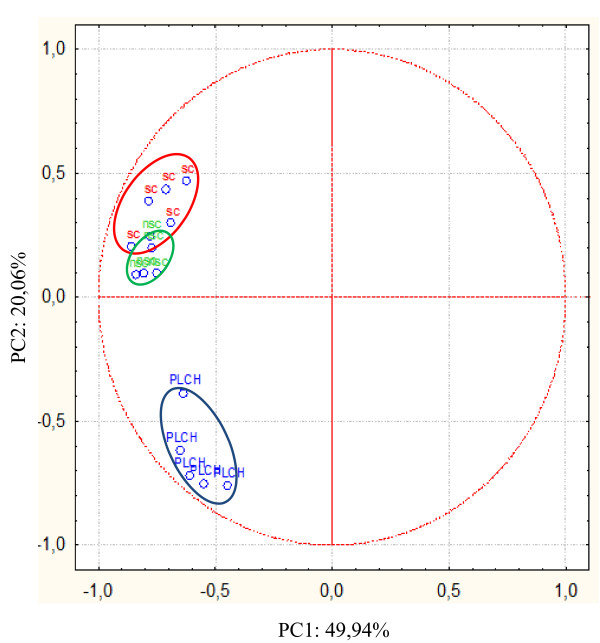
**PCA analysis**. The symbols represent the PC of the maps of spots expressed differently in BAL of PLCH patients and controls. The red ellipse shows the PC distribution of spot maps of smoker controls, the green ellipse shows that of non-smoker controls and the blue ellipse that of PLCH patients.

Multivariate statistical analysis by PCA was used to examine global trends in protein expression in BAL of PLCH patients and non-smoker and smoker controls. These samples were grouped according to the variance of their protein expression (%V) and their spatial distribution is shown in Figure 7. The first principal component (PC1) explained 49.94% of the variance and the second (PC2) explained a further 20.06%. PCA showed that PLCH and control samples clustered in distinct groups along the PC2 axis. In the control cluster, there were two other distinct groups very close to each other: those of non-smoker and smoker controls.

## Discussion

BAL protein expression analyzed by 2DE in a population of PLCH patients was compared with that of control samples. Bioinformatics analysis identified a wide range of spots expressed differently in BAL of PLCH patients with respect to BAL of healthy controls. The effect of cigarette smoking on the expression of some proteins was also evaluated, comparing BAL protein patterns of smoker and non-smoker controls.

### Population

The clinical, immunological and functional features of our PLCH patients indicated prevalently obstructive lung function deficit, increased BAL CD1a+ cells together with neutrophilia and eosinophilia, in line with the literature [1,2].

### 2DE

Proteomic analysis of BAL revealed 59 spots expressed with quantitative differences and 9 spots expressed with qualitative differences in BAL of PLCH patients with respect to controls. The proteins identified from these spots are involved in specific biological mechanisms (inflammation, immunity, oxidative stress, protease-antiprotease balance, cell proliferation, fibrosis) potentially implicated in the pathogenesis of PLCH. Some of these proteins need to be studied in detail, as they could be useful diagnostic or prognostic biomarkers.

Two proteins never described in BAL were identified *de novo*: serpin B3 and plastin 2. The first, up-regulated in smokers and higher (with borderline significance p = 0.05) in PLCH than controls, is a member of the family of protease inhibitors involved in cell survival and associated with lung cancer [24]. The second protein, plastin 2, member of a large family of actin filament cross-linkers, was down-regulated in PLCH patients with respect to smoker controls. Plastin 2 triggers immune response, cell migration, proliferation and cell-adhesion [25] and its role in actin cytoskeleton rearrangement and T-cell activation is crucial. Another function of plastin 2 is protection against TNF-cytotoxicity [26]. As cigarette smoke may induce production of tumor necrosis factor-alpha (TNF-α) by alveolar macrophages [27], up-regulation of PLSL2 in BAL of smokers may have a protective role against this pro-inflammatory cytokine. Interestingly in our PLCH patients this mechanism was down-regulated.

The results of our proteome analysis of PLCH BAL suggested the involvement of some immunoinflammatory pathways in its pathogenesis, which remains obscure. For example, the profibrotic effect of certain proteins could play a key role in development of PLCH. Pigment epithelium derived factor (PEDF) is a protein known to be involved in fibrogenesis. In our study PEDF was significantly higher in BAL samples of PLCH patients than smoker and no-smoker controls. This protein is an endogenous anti-angiogenic factor [28] implicated in a variety of diseases in which angiogenesis is critical, such as non-small cell lung cancer and IPF [28-31]. Immunohistochemical studies on IPF located PEDF in fibroblastic foci and areas of active matrix synthesis, where vascular density is low [31]. Recent research indicates that PEDF can be regarded as a TGF β1-mediated profibrotic agent [32]. These findings suggest that PEDF may be implicated in the regulation of vascular and fibrotic damage occurring in PLCH.

The role of angiogenesis in the pathophysiology of PLCH is controversial. Little data is available about neovascularization in PLCH [33]. Senechal et al. recently reported that PLCH lesions were sites of neoangiogenesis and tissue remodelling [34], whereas an immunohistochemical analysis by Zielonka *et al. *indicated that PLCH granulomas are connected with areas of extensive neoangiogenesis in which interleukin 1 alpha (IL-1α) and TNF-α are over-expressed [35]. In contrast to these lung tissue results, it has also been found that serum from PLCH patients inhibited angiogenesis [35]. Our study demonstrated that several proteins implicated in vascular remodelling were up-regulated in BAL of PLCH patients versus controls. Annexin A3, for example, is a calcium- and phospholipid-binding protein involved in angiogenesis as well as in cell proliferation, motility, invasiveness and signaling pathways [36,37]. This protein, up-regulated in PLCH patients with respect to controls, is reported in the literature to be over-expressed in lung adenocarcinoma associated with metastases [38]. Its multiple functions in PLCH pathogenesis warrants further investigation.

Our study suggests an imbalance between protease and anti-protease with consequent proteolytic-mediated lung damage potentially involved in the pathogenesis of PLCH, confirming previous observations [39]. In fact, we found a great abundance of proteolytic fragments of plasma proteins in BAL of PLCH patients, suggesting increased proteolytic activity. In particular kininogen 1 and haptoglobin proteolytic fragments were more highly expressed in BAL of PLCH patients than BAL of controls. An increased anti-proteolytic activity was found expressed by the significant increase of five isoforms of alpha 1-antitrypsin in BAL of PLCH patients with respect to smoker and/or non-smoker controls [39].

Several studies have analyzed smoke-induced oxidative stress in normal subjects but little data is available on the potential role of oxidation in PLCH [40]. Glutathione peroxidase 3 is an antioxidant protein with a protective role against cigarette smoke-induced lung inflammation [41]. It protects cells and enzymes against oxidative damage by catalyzing the reduction of hydrogen peroxide, lipid peroxides and organic hydroperoxide by glutathione [41]. Interestingly, in our research this protein was significantly higher in smoker than non-smoker controls but almost absent in BAL of PLCH patients (who were all smokers). It should be investigated if there is a defective production or/and an increased consumption in PLCH, as it has been demonstrated that oxidative stress is generally higher in PLCH patients than smoker controls [40]. Thioredoxin was another antioxidant protein down-regulated in BAL of PLCH patients with respect to smoker controls. It plays a protective role against cigarette smoke-induced lung oxidative damage [42,43] and reacts against reactive oxygen species (ROS) and other free radicals which are considered causative factors of smoke-related diseases in humans [44]. Thioredoxin counteracts Th2-driven airway inflammation by suppressing local production of macrophage migration inhibitory factor (MIF), irrespective of systemic Th1/Th2 immune modulation [45]. Interestingly, THIO is not only down-regulated in PLCH but also in idiopathic pulmonary fibrosis (IPF) [46].

Polymeric immunoglobulin receptor is a transmembrane protein involved in mucosal immunity (mediating transcytosis of polymeric IgA and IgM) [47,48]. This protein was significantly down-regulated in BAL of PLCH patients with respect to controls. Stress, smoking and inflammation can modulate PIGR production through TNF-α and interleukin-1β (IL1β), allowing translation of systemic inflammatory signals into mucosal immune responses [49], this mechanism seems to be compromised in PLCH. Recruitment of Langerhans cells in the lungs during exposure to smoke may induce T-helper 1 and T-helper 17 responses in CD4 T cells. Th17 cells produce interleukin 17 (IL17) that enhances secretion of CCL20, a chemoattractant for dendritic cells and matrix metalloproteinase 12 from lung macrophages [50,51]. Th17 and Th1 also promote PIGR activity by production of IL-17 and IL-1 [47]; this mechanism creates feedback that induces inflammatory cell recruitment and lung destruction [47]. The large quantity of Th17 in smoke-exposed lungs may therefore explain the high levels of PIGR required to amplify the mucosal immune response in BAL of smokers. This protein showed a different pattern in PLCH than in healthy smokers being decreased in PLCH, although PLCH patients were all smokers, suggesting a possible pathogenetic (not smoking related) role. PIGR, Th1 and Th17 immune responses should be deeply investigated in PLCH.

Another interesting protein potentially involved in PLCH pathogenesis could be annexin A1, a cell mediator of the anti-inflammatory action of glucocorticoid [52] that inhibits neutrophil extravasation [53]. The inflammatory environment induced by smoking is associated with increased epithelial permeability to neutrophils, macrophages and myeloid dendritic cells [4,42,54]. Complete loss of ANXA1 found in BAL of PLCH patients may lead to reduced response to steroids, over-recruitment of neutrophils in the lungs and loss of negative feedback for extravasation.

### PCA

In this study, PCA and analysis of the patterns of proteins differently expressed enabled us to distinguish our BAL samples into three groups (PLCH patients and smoker and non-smoker controls), which was one of our aims. Very high reproducibility was observed between BAL samples and distinct expression patterns in the three groups. Conducting multivariate analysis by PCA, we distinguished three groups in relation to the PC2 *y*-axis, and observed that non-smoker and smoker controls were both in the upper part of the graph, close together. This suggested that their patterns of protein expression were more similar to each other than to the PLCH group, despite the fact that they, too, were clearly separated, not only suggesting similar characteristics but also that exposure to cigarette smoke induced a modest change in the pattern of protein expression in BAL (smokers versus non-smokers). The position of the PLCH group on the opposite side of PC2 with respect to controls confirmed that the disease group had a protein profile different from that found in a condition of health (Figure 7).

In conclusion, proteomic analysis of BAL from patients with PLCH and smoker and non-smoker controls distinguished proteins up- and down-regulated in the disease differently expressed from smoker controls and than disease-related. Among these proteins there were PIGR and thioredoxin. The observation that certain proteins, over-expressed in PLCH patients, are also elevated in IPF suggests common pathways for the development of lung fibrosis [55]. Our proteomic study also indicates that oxidative stress, proteolysis and angiogenetic factors may be involved in the pathogenesis of PLCH, although further studies are needed also to assess the impact of other agents including pollution. Our future aim will be to further investigate the functions of the proteins of interest, their potential modifications induced by local damage (i.e. oxidation and proteolysis) and to validate the present results on a larger patients population.

## Abbreviations

PLCH: pulmonary Langerhans cell histiocytosis; BAL: bronchoalveolar lavage; HRCT: high resolution computed tomography; PFT: pulmonary function test; 2DE: two-dimensional electrophoresis; MS: mass spectrometry.

## Competing interests

The authors declare that they have no competing interests.

## Authors' contributions

EB corresponding author, design study, CL analysis and acquisition of data, PR study coordination, elaboration of results, LB critical revision, analysis of results, BM elaboration of results, critical revision, AP analysis, design study, JMQ conception and design of the study. All authors read and approved the final manuscript.
